# Cytotoxic Effects of two Iranian Scorpions *Odontobuthusdoriae* and *Bothutus saulcyi* on Five Human Cultured Cell lines and Fractions of Toxic Venom

**Published:** 2012

**Authors:** Amir Ahmad Salarian, Amir Jalali, Abbas Zare Mirakabadi, Hossein Vatanpour, Farshad H. Shirazi

**Affiliations:** a *Department of Toxicology, School of Pharmacy, Shahid Beheshti University of Medical Science, Tehran, Iran.*; b *Department of Toxicology and Pharmacology, School of Pharmacy and Toxicology Research Center, Ahvaz Jundishapur University of Medical Sciences, Ahvaz, Iran.*; c *Razi Vaccine and Serum Research Institute, Karaj, Iran.*; d *Pharmaceutical Sciences Research Center, Shahid Beheshti University of Medical Sciences, Tehran, Iran.*; e *Student Research committee, School of Pharmacy, Shahid Beheshti University of Medical Sciences, Tehran, Iran.*

**Keywords:** Scorpion, *Odontobuthus doriae*, *Bothutus saulcyi*, 1321N1

## Abstract

Scorpion venom toxicity is of major concern due to its influence on human activities and public health. We investigated the *in-vitro *process of cell death caused by two Iranian scorpions *Odontobuthus doriae *and *Bothutus salceyi *venom on human cell lines. The aim of this study was to provide further information about triggering cell death and suggestion of methods for the elimination of unwanted cells such as tumor cells.

The cytotoxicity and apoptosis induced by effect of scorpion venoms on five established eukaryotic cell lines are analyzed on different human cell lines. All cultured cell lines were incubated with varying doses of scorpion venom for 24 h at 37°C. Control culture was treated with an equal amount of SFM. The percentage of cell survival was measured using the 3-[4,5-dimethylthiazol-2-yl]-2,5-diphenyltetrazolium (MTT) colorimetric assay. Our data demonstrated that *Bothutus saulcyi*, does not show cytotoxic effect on any of the used cell lines. *Odontobuthus doriae*, however, has resulted a dose dependent cytotoxic effect with maximum at 1 ug/mL on 1321N1 glioma like cell line. Then the cytotoxic venom of *O. doriae *was fractionated using Sephadex G50 gel chromatography. The toxic fractions on mouse used to Cytotoxicity assay on 1321 N1 cell line and data demonstrated that, the fraction F3 showed a dose dependent Cytotoxicity assay. Further studies to explode the mode of action of these venoms are recommended and purification of the toxic fraction should be done.

## Introduction

Among the venomous animals are the scorpions. During about 400 millions years, they have successfully developed a large variety of bioactive peptides (1). Scorpions are widely spread around the world. There are about 1500 species of scorpions (2) from which approximately 25 species are dangerous to humans (3) especially for children and elderly (4). Thus, envenomation by scorpion remains a serious health problem especially in tropical countries (5). The Iranian scorpion fauna consists of over 44 named species from 23 genera in two families of Buthidae and Scorpionidae. In Iran, the same as other parts of the world, there are a few known species of scorpions responsible for severe envenoming. Of these, at least seven species have been implicated in envenoming of human which considered important medically (6). Among the most dangerous scorpions of Iran are those that belong to the family of Buthidae, such as *Odontobuthus *and *Buthus *(7). Most of the scorpion toxins have been isolated from the venoms of scorpions in the family Buthidae. The scorpion *Buthotus saulcyi *belonging to the Buthidae family is widely founded in the western region of Iran, but no published articles has been found to date on its venoms cytotoxicity effect (8). The genus *Odontobuthus *has three species: *bidentatus*, *doriae *and *odonturus*. Specifically, *Odontobuthus doriae*, the yellow scorpion, can be found in the central and southern parts of Iran. Its sting can cause various effects ranging from local pain, inflammation and necrosis to muscle paralysis, which might be deadly for children (9, 10). Scorpion venoms are composed of a variety of biologically active components such as enzymes, peptides, nucleotides, lipids, mucoproteins, biogenic amines and other unknown substances (11). The best studied group of scorpion venom components comprises the neurotoxins with polypeptides that recognize ion channels and receptors in excitable membranes (12).

The biological effects of scorpion stings are mainly due to the presence of low-molecular-weight proteins in the venom that exert powerful effects on excitable cells (9). Although the main effects of scorpion venom are likely to be due to toxins that affect the opening of ion channels in nerve and muscles, the mechanism by which the venom from the Iranian yellow scorpion *O. doriae *causes its neuromuscular effects *in-vitro *is not fully understood (9). Membrane channel blockers are known to control certain cellular behavior in the metastatic cascade (13) and also play a key role in cellular mitogenesis (14). This hypothesis may arouse the curiosity to study the antiproliferative and cytotoxic potentiality of scorpion venom (15). This study was carried out to investigate on the cytotoxicity of these venoms on five different human cell lines.

## Experimental

Venom preparation: the freeze dried venom was dissolved in distilled water and placed in dialysis bag and dialyzed against distilled water at 4 °C for 48 h. The venom solution was then centrifuged at 15000 rpm for 15 min and supernatant was collected for experiments. Protein concentration determination: Protein concentration was assayed in triplicate according to Bradford (1976) as modified by BioRad Inc., using bovine gamma globulin as a standard. Enzyme specific activities and all other analyses were based on these protein concentrations. Standard curve was drawn using bovine serum albumin (BSA) for any new plate or assay. Cell lines: Five different human cell lines have been used in this investigation. The HEK293 cell line that is derived from human embryo kidney and is transformed with sheared human Ad5 DNA which is sensitive to human adenoviruses and adenovirus DNA, OV2008 (Ovarian cell line), 1321N1 (Glial-like cell line derived from a human brain astrocytoma), HEPG2 (derived from liver tissue of a 15-year-old Caucasian male), and A549 (established from an explanted lung tumor which was removed from a 58-year-old Caucasian man in 1972). All cells were propagated in EMEM (HEPG2, OV2008 and HEK293) or DMEM (1321N1 and A549) medium (Sigma, St. Louis, MO, USA) supplemented with 10% fetal bovine serum (FBS), 2mM L-glutamine, and the antibiotics penicillin (10,000 units/L) and streptomycin (100 mg/L). The cell lines were incubated in flasks as monolayers and cultured at 37°C with 5% CO2 in a fully humidified atmosphere. Cytotoxicity assay: For the purpose of examining the effects of scorpion venoms on cell growth, a colorimetric 3-(4,5-dimethylthiazol-2-yl)-2,5-diphenyl tetrazolium bromide (MTT) assay was used. This assay is based on the cellular conversion of a tetrazolium salt (MTT) into a formazan product that is easily detected using a 96-well plate reader. Venoms were initially diluted in PBS and then serial dilutions were prepared using serum free cell culture medium in 96-well microtiter plates (Costar). Cells (1.5 × 104 to 0.5 × 105 cells well) were added into wells containing various concentrations of venoms (0.125, 0.25, 0.5, 0.75,1, 5, 10 μg/mL), and grown for 24 h. At 24 h, cell culture medium in each well was replaced with 200 μL of medium containing 0.5 mg/mL MTT, followed by incubation at 37°C for 3 h. DMSO was added into each well and the optical density at 570 as the base and 650 nm as the test was measured by a microplate reader (Bio-Rad 550). In each experiment, six wells were used, and experiments were repeated three times. 50 ug/mL of Cisplatin was used as a cytotoxic agent for control group.

Fractionation Procedure: 500 mg of the crude venom of *O. doriae*, was dissolved in 10 mL of 0.1 M ammonium acetate in water (pH 8.6). The solution was centrifuged for 30 min in 5000 rpm and the non soluble part removed and fractionation carried out with clarified sample in Sephadex G50 column (210 × 3.5 cm) in room temperature. Flow rate was 1 mL/min. Absorbance was measured at 280 nm and the content of tubes of each curve was mixed. The fractions were dialyzed in 1500 D cut off for 24 h and lyophilized at -50°C. Protein content determined as described above. Lethality tests and LD50 determination in mice: The mice (white, of both sexes, weighing 20 g) used in this research were treated in compliance with the US Public Health Service policy on human care and the use of animals. The mouse lethality of various protein fractions was measured using Muench and Reed method after IV injections to mice.

Toxic fractions were determined and used for Cytotoxicity assay on 1321N1 cell line. Statistical Analaysis: GraphPad Prism 3.02 version software was used for statistical analysis. One-way ANOVA and Newman-Keuls Multiple Comparison Test were used for analyzing of data.

## Result and Discussion

The dried scorpion whole body has been widely used in traditional Chinese medicine based on the Chinese herbalism ‘combat poison with poison’. Even at the present time, it is still used as a drug to treat neurological symptoms such as painful headaches, convulsion, seizures, swellings and paralysis (15).

The present study was conducted to evaluate the antiproliferative activity of *O. doriae *and *B. saulcyi *venoms on human cell lines. The antiproliferative and cytotoxic effect of venoms were supported by cell count using MTT assay. At longer time points, 48 h, the antiproliferative effect was reduced (unpublished data), which might be due to venom denaturation by the presence of temperature in aqueous solutions. That is why a 24 h exposure was used to determine the cytotoxicity of venom in most of experiments. 

**Figure 1:**
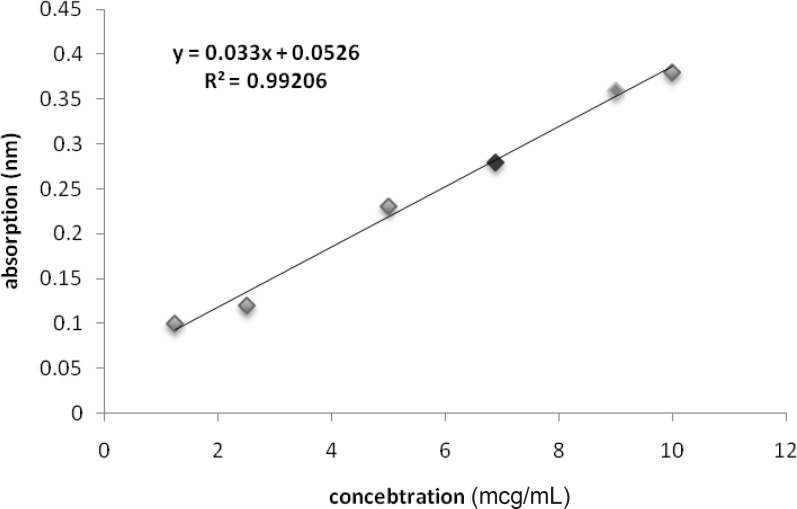
Calibration curve for protein concentration of solved venom in culture medium (Red spot is for O.Doriae and Green spot is for B.Saucyi).

The Bradford Protein concentration determination showed that absorption of the sample was 0.36. It means that the concentration of the sample was 9 ug/mL. this data was used as standard for the next steps (Figure 1).

**Figure 2:**
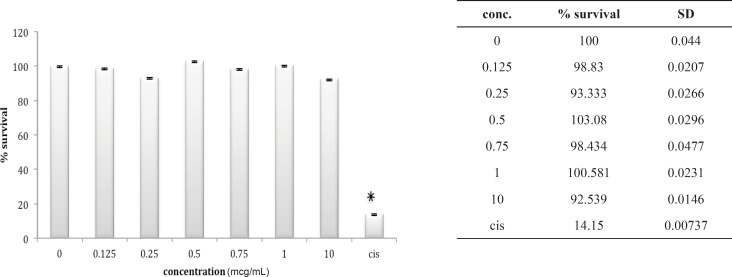
Cytotoxic effect of B.Saulcyi on HEK293 cell

Although HEK cell line is very much sensitive to the cytotoxic agents as is shown for cisplatin in our study, but seems to be very much resistant to either venoms. In case of *B. saulcyi *venom, a kind of resistance to the cytotoxicity effect is apparent for the HEK cell line (Figure 2).

**Figure 3:**
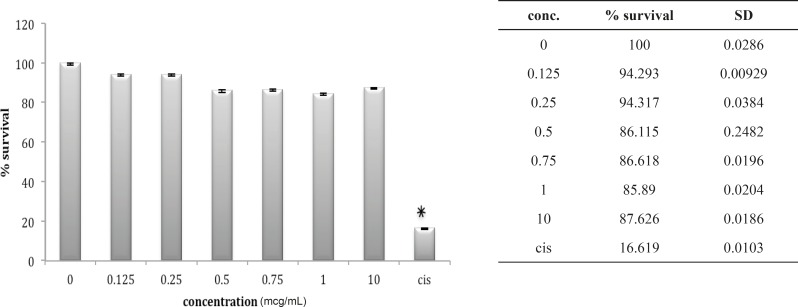
Cytotoxic effect of O.Doriae on HEK293 cell

 In fact, except for a minor and not significant decrease in cell number after exposure to 0.25 ug/mL of venom, no significantly reduction in cell population is shown after exposure to *B. saulcyi *venom. This cell line was not sensitive to *O. doriae *either. There is no significant reduction in HEK cell count in the presence of different doses of *O. doriae *venom (Figure 3).

**Figure 4:**
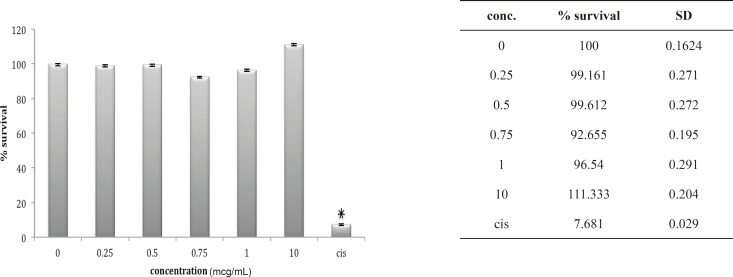
Cytotoxic effect of B.Saulcyi on OV2008 cell line

**Figure 5:**
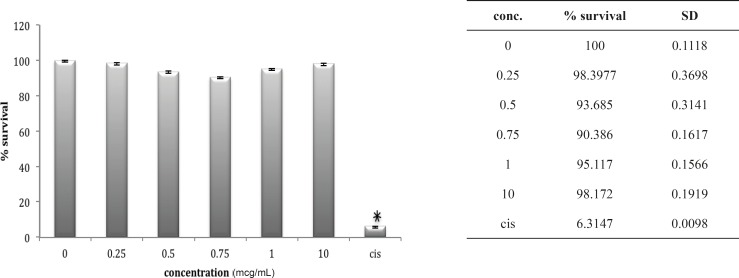
Cytotoxic effect of O.Doriae on Ov2008 cell line

Three other cell lines, OV2008, A549 and HepG2, look very much the same after 24 h *in-vitro *exposure to these venoms. The OV2008 cell line was not sensitive to either *O. doriae *or *B. saulcyi *venoms, with no significant difference between control group and groups that exposed to different doses of venom (Figures 4 and 5). 

**Figure 6:**
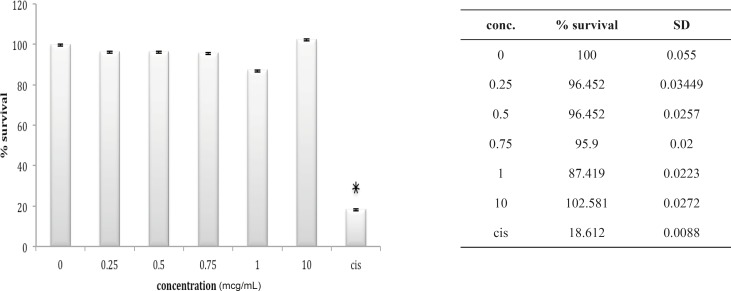
Cytotoxic effect of B.Saulcyi on A549 cell line

**Figure 7:**
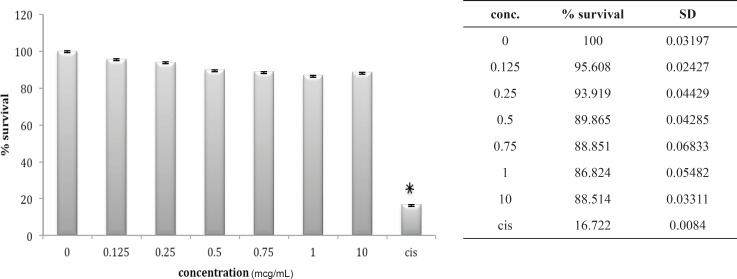
Cytotoxic effect of O.Doriae on A549 cell line

**Figure 8:**
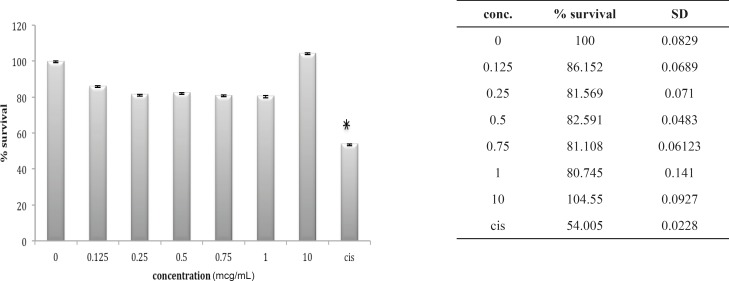
Cytotoxic effect of O.Doriae on HepG2 cell line

**Figure 9:**
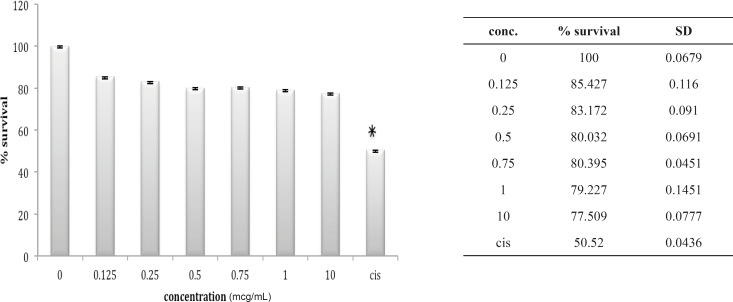
Cytotoxic effect of B.Saulcyi on HepG2 cell line

A549 cell line has neither shown any sensitivity to *O. doriae *or *B. saulcyi *after *in-vitro *exposure (Figures 6 and 7). The same story is truth for the HepG2 cell line (Figures 8 and 9) in spite of its sensitivity to the cytotoxic agent of cisplatin.

**Figure 10:**
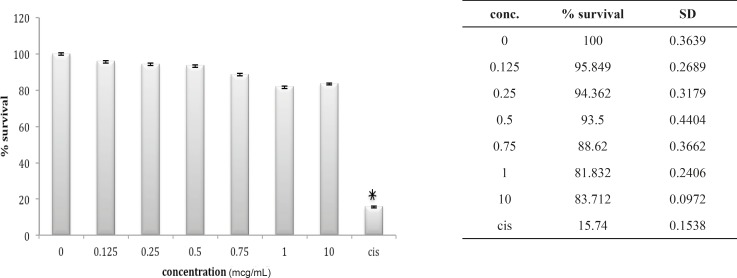
Cytotoxic effect of B.Saulcyi on 1321N1 cell line

**Figure 11:**
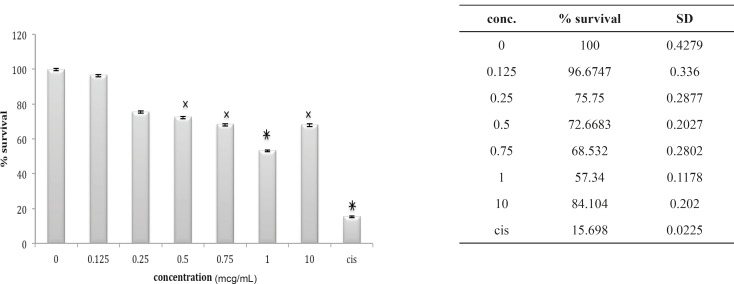
Cytotoxic effect of O.Doriae on 1321N1 cell line

The 1321N1 cell line did not show sensitivity to *B. saulcyi *venom (Figure 10) but it was, sensitive to *O. doriae*. Among the different doses tested at different cell lines 1 ug/mL venom of *O. doria *at 24 h was found to produce a marked inhibition on the 1321N1 proliferation with a p value of < 0.001 (Figure 11).

**Figure 12:**
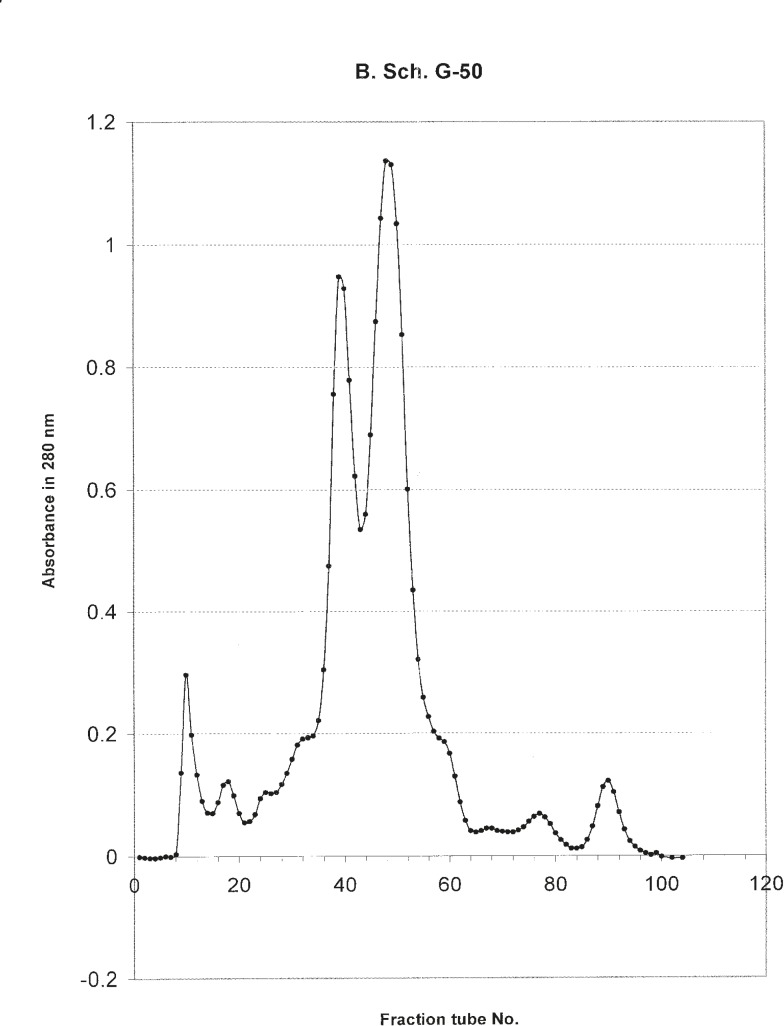
absorption of different tubes gathered from G50 Sephadex gel filtration

The hallmark of carcinogenesis is uncontrolled cellular growth and proliferation. In normal cells, cell proliferation and DNA replication is monitored by cell cycle check points and apoptosis. We found that from five different cell lines in our Cytotoxicity assays, the venom of *O. doriae *has only decreased the absorbance of MTT dye on 1321N1 cell line. Based on Cytotoxicity results that showed *O. doriae *crude venom was toxic on 1321N1 cell line, the venom was fractionated for determining of the toxic fraction. Fractionation with Sephadex G50 gel chromatography, isolated six fractions of *O. doriae *crude venom. (Figure 12)

**Figure 13:**
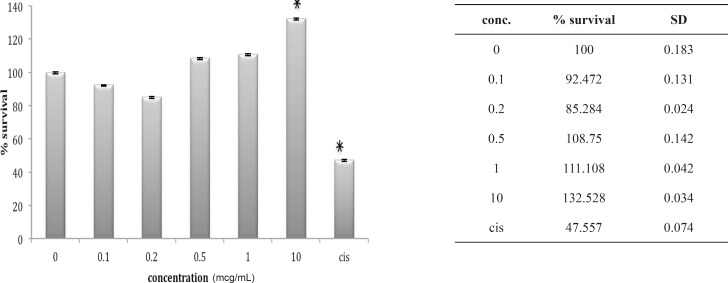
Cytotoxic effect of fraction F4 on 1321N1 cell line

**Figure 14:**
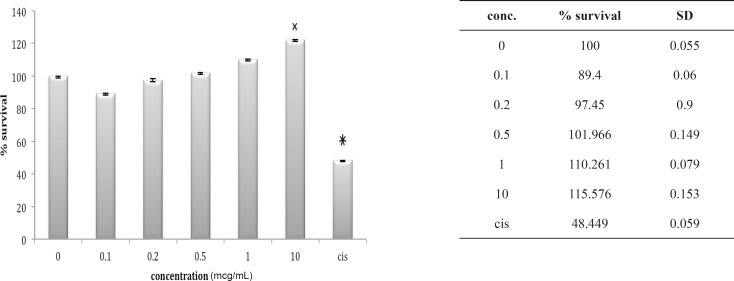
Cytotoxic effect of fraction F6 on 1321N1 cell line

**Figure 15:**
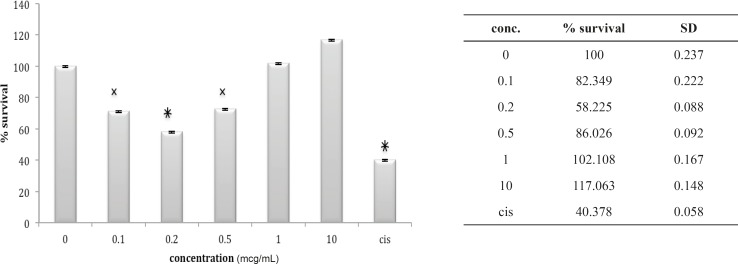
Cytotoxic effect of fraction F3 on 1321N1 cell line

Lethality test showed that fractions F3, F4 and F6 were toxic in mouse. (Table 1) these fractions used for further Cytotoxicity assay on 1321N1 cell line. The Cytotoxicity result showed that fractions F4 and F6 had no toxic effect on 1321N1 cell line (Figure 13 and 14) but fraction F3 was toxic on this cell line at 0.1 ug/mL (p < 0.001) (Figure 15). Scorpion venom contains a vast number of substances with different biomedical and pharmacological activities. Biochemical and pharmacological studies during the past decades have resulted in numerous polypeptidyl toxins isolation and characterization from crude scorpion venom, including long chain toxins with 60-76 amino acids which interact with Na^+^ channels (16), short-chain peptides with 28–42 amino acids which were deemed to be selective for K^+^ channel blockers, and chlorotoxin, a 36 amino acid peptide, that specifically binds to small conductance Cl channels (17).

**Table 1:** Recovery percent of three toxic fractions from dialyzed venom

**Fraction type**	**Recovery%**
F3	0.24
F4	0.14
F6	0.21

 Up to now, toxins isolated from *O. doriae *that are active on voltage-gated Na channels and potassium channels have been studied widely (18, 19) in glioma cells. These channels play key roles in the processes of glioma cell proliferation, swelling, migration, invasion (20-22). Blockage of these channels by this venom may therefore have a negative influence on gliomas› proliferation. Several groups have also hypothesized that the movement of ions through ion channels facilitate the growth and dissemination of glioma cells (23-29). Glioma cells display an up regulation of chloride, potassium, sodium, and aquaporin channels which are not found in normal astrocytes (30-36). These data suggest that mislocalization of K channel proteins to intracellular compartments is a characteristic of glioma cells. It may thus hypothesis that the unique *in-vitro *cytotoxicity of *O. doriae *venom on 1321N1 glioma cells among all of above studied cell lines, is due to the presence of the plenty of vital ion channels in this cell line, and the blocking effects of the scorpion venom components on these channels. Further studies to investigate on these mechanistically hypothesis are recommended.
